# Knighthoods for Hospital Secretaries

**Published:** 1916-11-04

**Authors:** 


### KNIGHTHOODS FOR HOSPITAL SECRETARIES.

The death of Mr. Melhado has produced a complaint in the newspapers, which is well worth recording as a sign of the times. A correspondent in the *Westminster Gazette* declares, and truly, that in the lay newspapers he noticed "only two very brief and wholly inadequate references" to the work of this remarkable man, who was also a responsible public official. After pointing out the rareness with which hospital secretaries receive any public recognition, the correspondent makes the following suggestion: "Our gracious King might well bestow the honour of knighthood now and then in their ranks. Physicians and surgeons of eminence are well paid and well recognised, yet many of them owe their success to their attachment to a great hospital that has been made great by a secretary's endeavours."

The point which emerges from this highly controversial statement of the facts is one which may legitimately be handled at the present time, when the profession of hospital secretary is taking on a new importance and stands on the threshold of a new era. The responsibilities which the war has brought to the hospital and nursing professions have given both a new importance in the life of the nation, and brought to both a new measure of public esteem. This has been fitly recognised in the case of the nurses by the bestowal of decorations, but the hospital secretary so far has received the esteem without the reward, the importance without the recognition. Such a state of affairs will not continue indefinitely, and the above proposal, coming, as it were, from without, is the hint of a change that cannot long be delayed. The profession of hospital secretary has had, like that of the actor, to fight a long time for recognition, and there are veterans among us who are certainly deserving of the honour proposed. But the position is more complicated than the correspondent in the *Westminster Gazette* seems to be aware. The veterans have earned their reward, but the profession as a whole has not reached the level to which individual members have carried it. It is still in a state of transition. It is still seeking how best to recruit itself. It still, as recent instances have reminded us, has sometimes to look outside its own ranks for persons fitted to do all that its most responsible positions demand. The discontent thus created is partly due to natural disappointment and partly to no less natural forgetfulness of the hard fact that a man may be fit for everything except promotion. Promotion cannot fall to those who have seniority and service only to recommend them, for a higher post demands not only experience. Its work is different as well as more onerous, and it must therefore fall to the man who has mark as well as method to his credit. The remedy for the present position is to improve the rates of pay sufficiently to attract those who from the start may hope to make the profession their life's work, with a legitimate prospect of reward. It has still to improve the *personnel* as well as the prospects of its younger members. The two sides of the question go hand in hand, and all we would here point out is that while individuals of eminence have certainly earned the reward proposed for them, authority may delay that reward until the necessary reforms have been carried out within its ranks in such a way as to secure that what is now true of individuals may become true of the profession as a whole. For it is one thing for a man to rise to the height of his responsibilities and another thing for those responsibilities to attract the man who will be able not only to shoulder them worthily, but to adorn his post so well as to make official distinction the natural and proper concomitant of it. At the moment our first duty is to raise the profession to the level attained by its best and ablest men.

